# Epidemiology of Endometriosis Awareness in Turkey

**DOI:** 10.7759/cureus.37536

**Published:** 2023-04-13

**Authors:** Bahar Yuksel Ozgor, Sena Azamat, Ezgi Gizem Berkay, Dilruba Türeli, Iclal Ozdemir, Secil Topaloğlu, Asiye Kocaturk

**Affiliations:** 1 Department of Obstetrics and Gynecology, Faculty of Medicine, Biruni University, Istanbul, TUR; 2 Department of Radiology, Cam and Sakura City Hospital, Istanbul, TUR; 3 Department of Basic Sciences, Istanbul Kent University, Istanbul, TUR; 4 Department of Obstetrics and Gynecology, Istanbul University, Istanbul Faculty of Medicine, Istanbul, TUR; 5 Department of Midwifery, College of Health Sciences, Istanbul Medipol University, Istanbul, TUR

**Keywords:** epidemiology and public health, turkiye, rates, symptoms, endometriosis

## Abstract

Introduction: Endometriosis is a chronic painful disease that affects the daily quality of life of individuals. Estimated rates show one in 10 women has endometriosis, although the actual prevalence is unknown. In this study, the impact of endometriosis prevalence and symptoms on women's lives in Turkey was questioned through a web-based questionnaire.

Methods: We utilized a version of the World Endometriosis Research Foundation (WERF) EndoCost tool, which was sent to applicants via social media. Data from women aged 18-50 years were analyzed.

Results: The results of 15,673 participants have been analyzed, and 2880 (18.3%) participants had endometriosis. Respondents with endometriosis reported urinary, neurological, and gastrointestinal disorders at statistically higher rates when compared to individuals without endometriosis diagnosis (54.2%, 84.5%, and 89.9% vs. 37.2%, 75.5%, and 81.1%, respectively; p = 0.001). Most respondents with endometriosis (80.1%) reported persistent fatigue and 21.2% of endometriosis participants reported feeling socially isolated related to their condition (p = 0.001). Of the participants with endometriosis, 63.2% mentioned that people did not believe their pain or symptoms and 77.9% experienced financial difficulties due to the cost of therapy. Of the participants with endometriosis, 46.0% reported that they had problems in their personal relationships, 28.3% had difficulties at work/school, and 7.4% were unable to attend class/work due to endometriosis-related symptoms.

Conclusion: Endometriosis is a chronic, underestimated disease that affects 18% of Turkish women of reproductive age. There is a need for guidelines to inform healthcare providers, population professionals, and patients. Societies and governmental health authorities must work together to resolve this public health issue.

## Introduction

Endometriosis is an estrogen-dependent, chronic, inflammatory disease diagnosed with the presence of endometrial glands and/or stroma outside the uterine cavity [1]. Beside from its physical effects, it can alter the quality of life, mental health, relationships, and sexuality. Around 10% of women will develop it at some point in their lives, and it typically affects them from menarche through menopause [2]. This chronic disease may affect all organ systems and may cause organ-related symptoms, including dysmenorrhea, dyspareunia, chronic fatigue, chronic pelvic pain, and subfertility, while only 20-25% of endometriosis carriers are asymptomatic. For patients with infertility, endometriosis prevalence increases by up to 30%, and for those with chronic pelvic pain, the prevalence increases by up to 45% [3,4]. Moreover, the delay in the diagnosis of endometriosis causes a loss of labor, a decrease in productivity, and excessive costs [5]. Most cases go undiagnosed due to the need for laparoscopy as the gold standard method for diagnosis, and not all cases have the opportunity for proper laparoscopic diagnosis. As expected, for all these reasons, the actual incidence of endometriosis in the population, including developed countries, is not known, and frequency studies are not adequate [6].

Previous studies from developed countries show women with endometriosis have lower annual incomes and higher short- or long-term workforce loss [7,8]. Furthermore, according to an Aotearoa New Zealand web-based survey, the education of 27% of school students is disrupted due to endometriosis-related symptoms. In addition, studies show that almost six years of delayed diagnosis contribute to 60% of lost productivity among endometriosis carriers [4,9].

There is a paucity of information about the prevalence of endometriosis in developing countries. This current study aims to evaluate the prevalence of endometriosis in Turkey and the impact of related symptoms on social, professional, medical, and daily life.

## Materials and methods

Endometriosis-related problems were surveyed in this cross-sectional analysis. For this purpose, the World Endometriosis Research Foundation (WERF) EndoCost tool was utilized [10]. However, it was altered to be used with a Turkish demographic and healthcare background, and the survey was sent to applicants through Google Forms (Google, Mountain View, CA) [11]. The ethical approval has been taken from Istanbul Medipol University Ethics Committee (E-10840098-772.02-4247). The primary method of recruitment was dissemination using social media platforms (Facebook and Instagram), including social media accounts of influencers who have followers from all over the country, accounts of authors and women’s support organizations, and countywide student clubs in universities, and by delivering to regions by nurses working in family medicine all around the country. The online information and a consent sheet were given on the first page of the survey. The survey was open for 10 weeks, from September 15, 2022, to November 2022. Participants who reported a histopathologic diagnosis of endometriosis on the questionnaire form were included in the endometriosis group. Exclusion criteria for responders were not being 18-50 years old, incompletely filling the questionnaires, participating from outside Turkey (foreign countries), or being diagnosed only by gynecologic examination or ultrasound but without a histological diagnosis. Because this survey was meant to identify the prevalence of symptoms and women with endometriosis diagnosis rather than test a hypothesis, a power analysis was not conducted.

Statistical analyses

The Statistical Package for the Social Sciences (SPSS) version 20 program (IBM Corp., Armonk, NY) was used for statistical analysis. Normality distribution was evaluated by the Kolmogorov-Smirnov test. Data were described using mean and standard deviation, medians, and interquartile ranges. Categorical values and proportions are expressed as percentages and were compared using the chi-square test or Fisher's exact test between groups. Pearson's and Spearman's rank correlations were used to evaluate the relationship between the participants and the population of cities.

## Results

**Figure 1:**
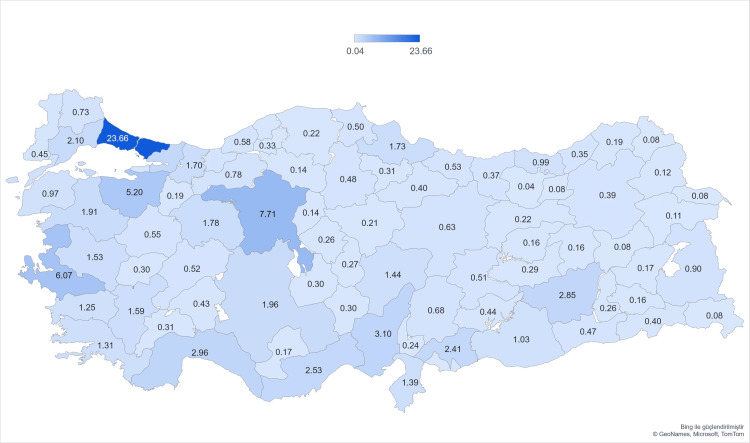
A high correlation was calculated with the official census data by province (correlation coefficient = 0.81, p = 0.001). ** Spearman's rho correlation analysis. Original figure.

A total of 16,304 surveys were completed, with 15,673 being included in the study. A total of 631 surveys were excluded due to incomplete data, participation from outside Turkey (foreign countries), and not being between 18 and 50 years old. Also, patients who reported being diagnosed with endometriosis but without a confirmed histologic diagnosis (256 participants) were excluded. The average age of responders was 30 years. Istanbul had the most respondents, followed by Ankara, Izmir, Bursa, and Antalya. There was a strong positive correlation between the distribution of respondents by province and the population of cities according to the Turkish Statistical Institute (Spearman's correlation coefficient = 0.81, p = 0.001) (Figure 1).

This survey had 15,673 participants, and 2880 (18.3%) were diagnosed with endometriosis. The mean number of days of menstrual cramps differed significantly between the two groups (p < 0.001). The average number of days of menstrual cramps was 2.7 ± 1.9 days for endometriosis participants and 2.1 ± 1.6 days for non-endometriosis participants. While 30.2% of endometriosis individuals reported menstrual cramps lasting three to six days, 3.1% of participants reported menstrual cramps lasting more than seven days. However, the percentage of individuals in the non-endometriosis group who had stated menstrual cramps lasted three to six days and lasted more than seven days were 20.3% and 2.5%, respectively (p < 0.05). Furthermore, there was a significant difference in the duration of menstruation and the number of pads used during menstruation (4.5 ± 2.1 days and 3.9 ± 1.8 pads vs. 3.9 ± 1.7 days and 2.8 ± 1.5 pads, respectively) (p < 0.001). The percentage of participants with endometriosis (1.3%) who reported quitting work/school due to pain was statistically higher than the non-endometriosis population (0.6%) (p = 0.001). The average number of working days lost per month by endometriosis and non-endometriosis participants was 1.3 ± 0.6 and 1.1 ± 0.7, respectively (p = 0.001). Among those diagnosed with endometriosis (2880 participants, 18.3%), 38 (1.3%) reported being unable to work owing to menstrual and/or continuous pain, while only 0.6% reported taking the day off for menstrual pain in the population with no surgical endometriosis diagnosis (p = 0.001) (Table 1).

**Table 1:** Comparison of demographic data of participants according to the diagnosis of endometriosis. * Mean ± standard deviation.

	Diagnosed with endometriosis	Not diagnosed with endometriosis	P-value
Number of participants	2880 (18.3%)	12,793 (81.7%)	
First menstrual age (years)*	12.6 ± 1.8	12.5 ± 1.9	0.864
How many days do your menstrual cramps last?			
1-2 days	2021 (66.7%)	9733 (77.2%)	0.001
3-6 days	914 (30.2%)	2556 (20.3%)	0.001
7+ days	93 (3.1%)	317 (2.5%)	0.022
Mean ± SD	2.7 ± 1.9	2.1 ± 1.6	0.001
How many days does your menstrual bleeding last?			
1-3 days	1081 (35.7%)	4197 (33.3%)	0.001
4-6 days	1780 (58.8%)	8104 (64.3%)	0.001
7+ days	167 (5.5%)	305 (2.4%)	0.001
Mean ± SD	4.5 ± 2.1	3.9 ± 1.7	0.001
How many pads do you use per day during your period?			
1-5	2861 (94.8%)	12,190 (96.7%)	0.001
6-9	99 (3.3%)	278 (2.2%)	0.001
10+	58 (1.9%)	138 (1.1%)	0.001
Mean ± SD	3.9 ± 1.8	2.8 ± 1.5	0.001
Employment status			
Working	2179 (75.7%)	8843 (68.8%)	0.001
Not working	349 (12.1%)	1772 (13.8%)	0.014
Student	292 (10.1%)	2047 (15.9%)	0.001
Quit work due to pain	38 (1.3%)	74 (0.6%)	0.001
Unable to work due to any health problems	22 (0.8%)	120 (0.9%)	0.373
The average number of days absent from work/school (per month)*	1.3 ± 0.6	1.1 ± 0.7	0.001

**Figure 2:**
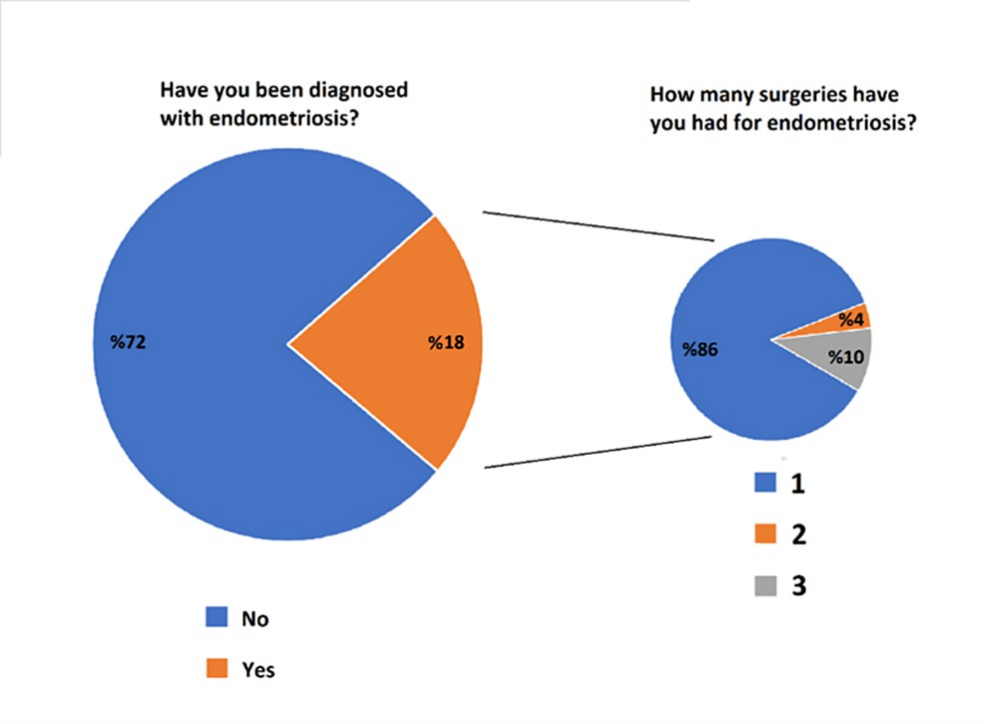
Endometriosis diagnosis and surgery statistics.

When endometriosis surgeries were questioned, 14% of participants with endometriosis reported having more than one endometriosis operation (Figure 2).

Impact of endometriosis

The proportion with difficulty getting pregnant was significantly higher in endometriosis patients (33.6 vs. 12.4%, p = 0.001). Brown spotting outside the menstrual cycle was significantly more common in endometriosis respondents (46.4 vs. 31.6, p = 0.001). Respondents with endometriosis reported urinary, neurological, and gastrointestinal disorders at statistically higher rates when compared to individuals without endometriosis diagnosis (54.2%, 84.5%, and 89.9% vs. 37.2%, 75.5%, and 81.1%, respectively; p = 0.001). Most respondents in both groups (73.4% of respondents with endometriosis and 66.1% without endometriosis) reported that abdominal bloating was the main gastrointestinal problem. Most respondents with endometriosis (80.1%) reported persistent fatigue, whereas only 59.5% of respondents without endometriosis reported fatigue as a problem. When asked, 21.2% of endometriosis participants reported feeling socially isolated related to their condition (p = 0.001) (Table 2).

**Table 2:** Comparison of complaints according to the diagnosis of endometriosis. * Mean ± standard deviation.

	Diagnosed with endometriosis	Not diagnosed with endometriosis	P-value
Number of participants	2880 (18.3%)	12,793 (81.7%)	
Difficulty getting pregnant	967 (33.6%)	1588 (12.4%)	0.001
Brown spotting outside of the menstrual period	1337 (46.4%)	4042 (31.6%)	0.001
Urinary system complaints	1562 (54.2%)	4758 (37.2%)	0.001
Nervous system problems (including migraine and peripheral nerve pains)	2434 (84.5%)	9656 (75.5%)	0.001
Persistent fatigue	2308 (80.1%)	7611 (59.5%)	0.001
Do you feel socially excluded?	610 (21.2%)	841 (6.6%)	0.001
Gastrointestinal system complaints	2589 (89.9%)	10,379 (81.1%)	0.001
Abdominal bloating	2114 (73.4%)	8462 (66.1%)	0.001
Abdominal pain	2037 (70.7%)	8544 (66.8%)	0.001
1 symptom	297 (10.3%)	1037 (8.1%)	0.001
2 symptoms	468 (16.3%)	2190 (17.1%)	0.262
3+ symptoms	1798 (62.4%)	7433 (58.1%)	0.001

**Figure 3:**
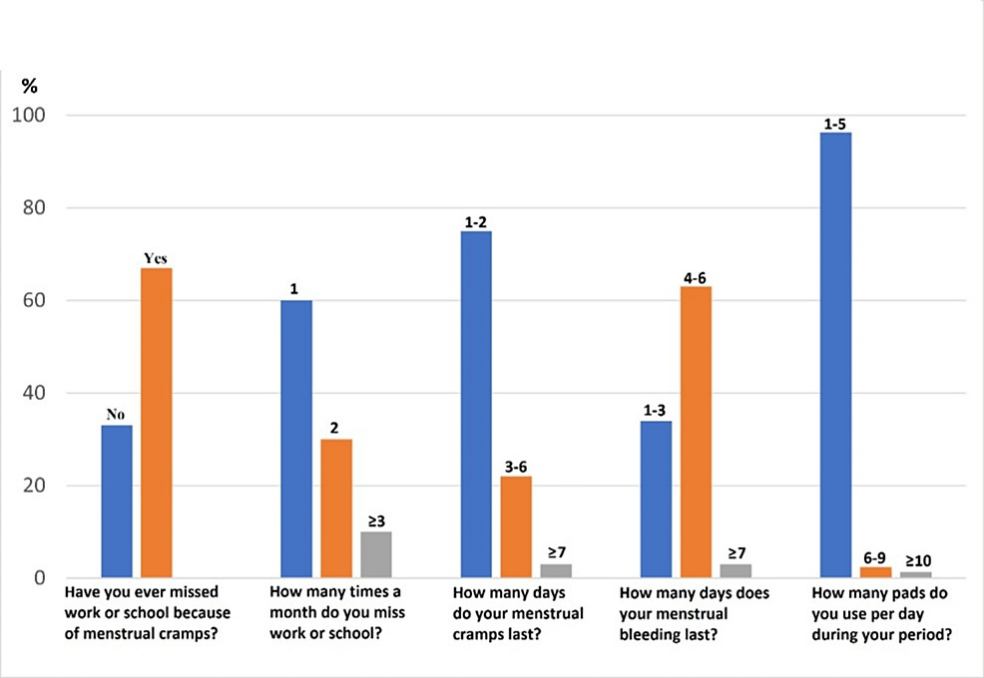
Duration of pain and bleeding during menstruation and the number of days lost from work and school due to these complaints.

**Figure 4:**
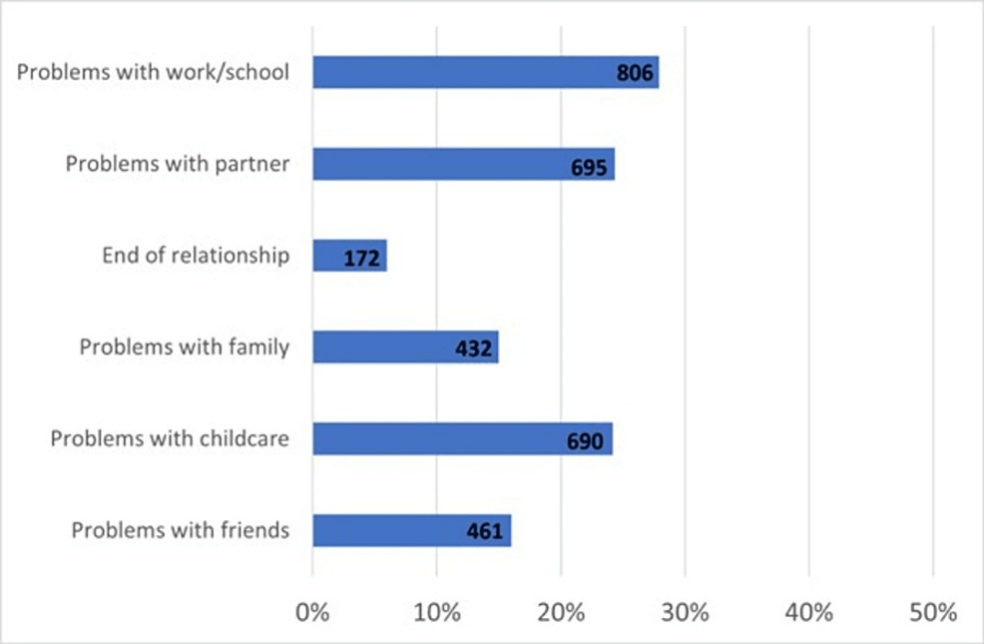
Problems in social relationships caused by endometriosis.

**Figure 5:**
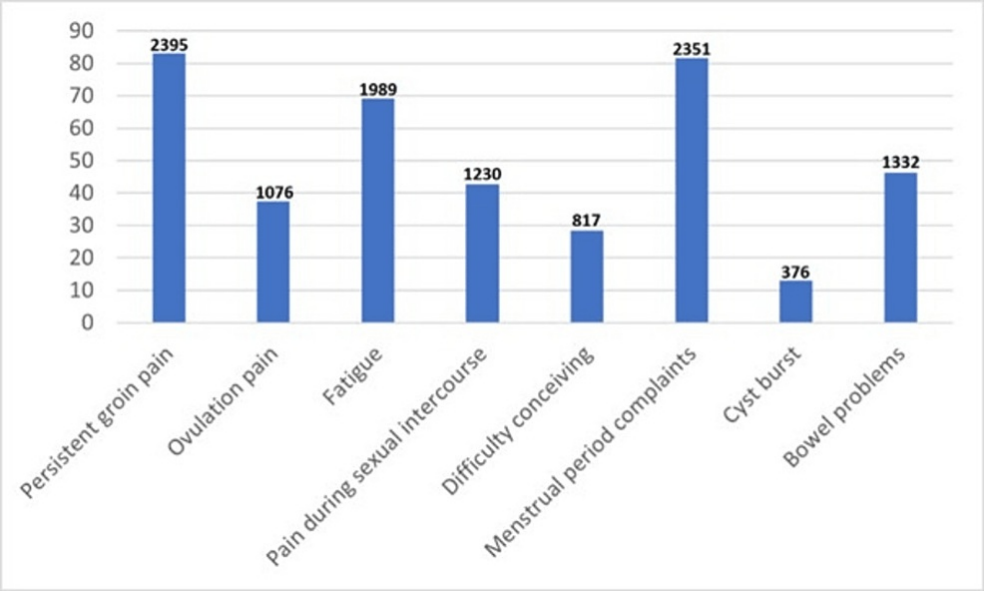
Complaints at the time of diagnosis.

According to 1820 (63.2%) participants with endometriosis, people did not believe their pain or symptoms. The average number of physician visits was four before diagnosis. The average duration from symptom onset and initial attendance visiting a clinician was eight months (IQR = 2-24 months). In patients with endometriosis, the average period between the initial hospital attendance and the diagnosis was four years (IQR = 1-5 years). Most participants with endometriosis (77.9%) experienced financial difficulties due to their therapy. A total of 1152 individuals with endometriosis reported work/school difficulties (28.3%), and 224 (7.4%) respondents could not attend class/work due to endometriosis-related symptoms. In addition, most respondents with endometriosis (46.0%) reported problems in personal relationships. The survey showed that the most common symptom that caused hospital attendance in the beginning was a complaint happening during the menstrual period, and persistent groin pain was the next most-common complaint. Painkillers were the most commonly used medical treatment for respondents with endometriosis (73.3%), while 15% needed more than three treatment options. The majority of respondents with endometriosis (66.9%) had at least one additional disease (Table 3). Data on symptoms caused by endometriosis are summarized in Figures 3-5.

**Table 3:** Data for participants with the diagnosis of endometriosis. * Median (IQR).

	Diagnosed with endometriosis (n: 2880)
Do you think people don't believe in your pain or symptoms?	1820 (63.2%)
How many times did you go to the doctor before the diagnosis?*	4 (3-5)
Time between your first complaint and first doctor visit (months)*	8 (2-24)
Time between your first doctor visit and diagnosis (years)*	4 (1-5)
Are endometriosis treatment costs straining your budget?	
Yes	2245 (77.9%)
No	515 (17.8%)
Treatment not started	120 (4.2%)
Problems with work/school	1152 (28.3%)
Inability to attend class/work	214 (7.4%)
1 problem	648 (22.5%)
2 problems	148 (5.1%)
3+ problems	122 (4.2%)
Problems in personal relationships	1324 (46.0%)
Problem with partner	708 (24.6%)
1 problem	449 (15.6%)
2 problems	409 (14.2%)
3+ problems	196 (6.8%)
First complaint at diagnosis	2880 (100%)
Complaints about menstrual period	1542 (53.5%)
1 symptom	1197 (41.6%)
2 symptoms	820 (28.5%)
3+ symptoms	679 (23.6%)
Type of medical treatment	2425 (84.2%)
Painkiller	2112 (73.3%)
1 medication/method	514 (17.8%)
2 medications/methods	842 (29.2%)
3+ medication/methods	432 (15.0%)
Additional diseases	1927 (66.9%)
Myoma uteri	216 (7.5%)
Anxiety/depression	187 (6.5%)
1 disease	528 (18.3%)
2 diseases	940 (32.6%)
3+ diseases	405 (14.1%)

## Discussion

According to our knowledge, this is the first high-attendee survey study to question the prevalence of endometriosis in Turkey. Studies from different regions of the world report that approximately one in seven or 10 women are diagnosed with endometriosis. The current study based on self-reported data revealed one in six of all women of reproductive age in Turkey has an endometriosis diagnosis. In this study, the numbers of the participants according to cities were correlated with the demographic distribution of populations in the cities according to data from the Turkish Statistics Institute [12]. This survey showed that most women with endometriosis differ from healthy women in terms of general health conditions, daily quality of life, personal relationship issues, and working and educational difficulties.

The most frequent complaints at the time of diagnosis were cyclic menstrual problems and persistent groin pain. Participants with a diagnosis of endometriosis stated they had painful menstrual days, more days of heavy bleeding, and sanitary pad numbers used during each menstruation with statistically significant higher numbers. According to a recent study about menstrual health and costs for Turkish women, around 77% reported difficulty reaching menstrual products [13]. Thus, when including social and financial parameters about women with endometriosis, even this little step is something that must be dealt with.

According to the study findings, women with endometriosis quit work and school due to being unable to manage pain, with 1.3% of participants with endometriosis stating that they could not work due to menstrual pain, while this rate was 0.6% in the undiagnosed group. In a study conducted in New Zealand, over 70% of respondents mentioned alterations in their employment status, such as losing their jobs, changing their profession, or reducing their working hours due to endometriosis-related symptoms [5]. In a study conducted on 931 women, 16% of participants declared that they had missed time in education due to endometriosis symptoms, and in their working careers, 48% had missed hours and 11% had lost their jobs [14]. As cultural variations might cause the differences in percentages, the loss of productivity is still two times higher in endometriosis patients when compared to the rest of the population.

There was a significant increase in symptoms for women with endometriosis, including difficulty getting pregnant, heavy bleeding, urinary disorders, migraines and peripheral nerve pain, and gastrointestinal complaints such as gas and bloating. Along with menstrual pain, bowel complaints, sexual intercourse problems, and persistent fatigue were the leading problems at the time of diagnosis. In a survey-based study conducted in the United States of America (USA), similar to the current results, the respondents revealed the most common symptoms were pelvic pain, gastrointestinal symptoms, fatigue, anxiety, and stress [15]. The rate of fatigue in the endometriosis population was 80.1%, which is very high when compared to the worldwide rate of 2.83% [16,17]. A study analyzing endometriosis-related complaints among Polish women found that pain and infertility were the most common symptoms [18]. In a study of the Israeli population, the incidence of infertility was reported as 36.9% while it was 33.6% in the current study, which is also consistent with the difficulty conceiving rates for women with endometriosis globally [19,20]. When it comes to infertility, pain causing partners to avoid intercourse should also be considered, along with endometriosis-related infertility mechanisms [21]. Also when analyzed together, it can be seen that not only menstruation-related symptoms are associated with endometriosis; thus, healthcare providers should be educated about possible relationships between general health problems and endometriosis.

In addition to physical problems, the social lives of women were also obstructed. With rates varying between 20% and 30%, the participants reported problems with their work/school life, partners, or children. Most importantly, 21.2% reported feeling socially excluded and 63.2% stated they were not believed about their complaints by their friends or family members, which is similar to results reported from Western countries [22]. The costs of endometriosis treatments have always been a topic of interest for studies all around the world. In a review questioning the economic burden of endometriosis, the authors found there were indirect costs related to absenteeism and presenteeism (lost productivity at work) [15]. In the current study, 77.9% of women with endometriosis reported that treatment costs burdened their budget, which included at least four doctor visits until diagnosis, an average of four years of delay until diagnosis, and loss of productivity. Additionally, 66.9% of women diagnosed with endometriosis stated they had at least one more medical condition, which means additional costs. These results are in parallel with the endometriosis burden in the USA and Australia [23,24]. Thus, provisions in healthcare policies, insurance coverage, and business plans are urgently required.

This study is a pioneering study in which not only participants with a diagnosis of endometriosis but also Turkish women without diagnosis reported complaints related to menstruation, and comparisons were made between the two groups. It is also a pioneering study among examples worldwide due to the number of participants and statistically reliable results.

The limitations of the current study firstly come from obtaining self-reported data. Also, due to the fact that endometriosis can only currently be diagnosed by being aware of symptoms, finding appropriate healthcare, and having surgery, there may be undiagnosed cases that remain in the non-endometriosis group. Third, while nurses working with family medicine specialists in rural areas also shared the surveys, some responses were obtained through online social media platforms, and there may be sample bias related to social media interests since social media may not be as widely used by the 40-50 age group. Fourth, while the study was designed to determine the current prevalence of the disease in Turkey reaching as many people as possible and, additionally, the results were consistent with national demographic statistics, still the subjects were not randomized. The survey used was a version of the WERF EndoCost tool, but it was not validated for the Turkish language.

## Conclusions

Endometriosis is a chronic, underestimated disease that affects at least 18% of Turkish women of reproductive age. Despite all the efforts and attempts to raise awareness about gender equality, the social and physical health of women with endometriosis is still depleted. There is a need for guidelines to inform healthcare providers, population professionals, and patients. This will not only provide a good quality of life but also will ensure the opportunity for new scientific studies about the diagnosis and treatment of the disease. Societies and governmental health sources must work together to resolve this public health problem.

## Appendices

**Table 4:** Descriptions of the data shown in Table 2 and Table 3.

Type of medication	First complaint at diagnosis	Problems with personal relationships	Problems at work/school	Gastrointestinal complaints
Hormonal medication	Severe menstrual cramps	Problems with my partner	I had to leave work-school	Diarrhea
Herbal medicine	Continuous groin pain	There were problems with my family	I changed section-field	Constipation
Yoga, sports, and physical therapy	Ovulation pain	It caused my relationship to end	I gave up on my dreams	Abdominal bloating
Vitamin supplements	Unrelenting fatigue	Made child care difficult	I missed my exams/classes	Gas
Painkillers	Peri-menstrual complaints	My relationship with friends was affected	I lost my job	Stomach ache
Diet change	Pain during sexual intercourse	My social life is over	I couldn't go to work	Anal pain
Alternative-complementary	Difficulty conceiving			Nausea-vomiting
	Cyst burst			Bleeding from the anus
	Bowel problems			Stomach ache
	Diaphragmatic endometriosis			
Additional diseases				
Rheumatism	Behcet’s disease	Celiac disease	Cardiac arrhythmia	Psoriasis
Sjogren’s disease	Ankylosing spondylitis	Irritable bowel syndrome (IBS)	Hypothyroidism	Sarcoidosis
Rheumatoid arthritis	Polycystic ovarian syndrome (PCOS)	Fibromyalgia	Epilepsy	Lupus
Hyperprolactinemia	Multiple sclerosis	Allergy-asthma		
